# High prevalence of depression among Iranian patients with first onset pseudoseizures

**DOI:** 10.4103/0019-5545.44749

**Published:** 2008

**Authors:** Alireza Farnam, Mohammad Ali Goreishizadeh, Sara Farhang, Fatemeh Abdolaliyan

**Affiliations:** Department of Psychiatry, Tabriz University of Medical Sciences, Tabriz, Iran

**Keywords:** Beck depression inventory, depression, pseudoseizure

## Abstract

**Background:**

Pseudoseizures are common and can pose a significant diagnostic and therapeutic challenge. Exploring the role of psychiatric disorders can be helpful. The aim of this study was to evaluate depression in patients with first onset pseudoseizure.

**Materials and Methods:**

Consecutive patients with pseudoseizure (DSM IV) in an emergency room (Tabriz, northwestern Iran) were evaluated with beck depression inventory (BDI) and were compared to age- and sex-matched patients with other neurologic disorders.

**Results:**

Forty-two patients with pseudoseizure (52% male; age: 25.7 ± 6.4) were compared to 50 patients with other neurologic disorders (55.1% male; age: 27.2 ± 5.7) by BDI. Marriage status and educational level were almost similar between two groups. The mean (±SD) score of BDI in patients with pseudoseizures and controls was 23.6 ± 7.4 and 14.14 ±10.5, respectively. Depression was more common and more severe among patients with pseudoseizures (96% vs. 60%).

**Conclusion:**

Depression is common among patients with pseudoseizure even in patients with first onset attacks.

## INTRODUCTION

Conversion disorder, as a subset of somatoform disorders defined in the diagnostic statistical manual of mental diseases (DSM) IV, is the occurrence of symptoms mimicking neurologic diseases like seizures or paralysis which are not intentionally produced and cannot be explained by any of known neurologic or general medical etiologies.[1] The etiology of nonepileptic nonorganic seizures, also referred to as psychogenic seizures, nonepileptic attack disorder or pseudoseizures is largely unknown.[2] A high prevalence is estimated for nonepileptic seizures about 3-0.3 to 4.6 per 100,000 subjects.[3,4] More over, the disorder is commonly of long duration and there is no common agreement on the best management.[5–7]

The patients are generally first seen and treated by neurologists and they can pose a significant diagnostic and therapeutic challenge. Accordingly exploring the role of psychiatric disorders in psychogenic non epileptic seizures (PNES) can be helpful. To the best of our knowledge, very little is known about the characteristics of these patients in Iran and there is a serious lack of information about conversion disorders and comorbid psychiatric problems of these patients.

In the present study, we compared the presence of depression between a group of patients with pseudo seizures and a group of neurologic visits in an emergency room. This study is of patients recruited to an emergency center, who had developed their symptoms for the first time.

## MATERIALS AND METHODS

Consecutive patients admitted to emergency ward of Imam University Hospital, Tabriz, Iran during September 2005-September 2006 who had a clinical diagnosis of pseudoseizures were considered for participation in this study. The diagnosis made based on DSM-IV criteria by the same trained physician after excluding any organic explanation by a neurologist. Patients were excluded lf they been diagnosed with sever somatic disease like recent myocardial infarction, sequel of stroke, drug addiction, and serious mental disorders.

Random patients from the same ward were enrolled to the study as control group and accepted the interview after receiving specific heath care service. The two groups were matches by age sex.

The study purpose was fully explained and patients provided written informed consent. Depression was evaluated in both groups by the Farsi version of Beck Depression inventory[8] by a trained interviewer blinded to the diagnosis of patients.

The beck depression inventory (BDI) is a 21-item self-report instrument that evaluates the severity of depression, with possible score ranging from o to 63.

Data were analyzed using the SPSS package (version 13). Continuous variables (BDI score, age) were compared using the student's *t*-test. Correlations between nominal variables were analyzed using chi-square test. Statistical testing was done at the two-tailed level of 0.05.

## RESULTS

Fifty patients with pseudoseizure participated in this study. Fifty patients with other neurologic disorders completed BDI as control group. The mean (±SD) age of the patients with conversion disorder (52% male) was 25.7 ± 6.4 years (controls: 55.1% male; 27.2 ± 5.7 years). Marriage status and educational level were almost similar between two groups.

BDI was self-completed in approximately 10 minutes. The mean (±SD) score of BDI in patients with pseudoseizures and controls was 23.6 ± 7.4 and 14.14 ±10.5, respectively (*P* < 0.0005). Depression was more common among patients with pseudoseizures (96% vs. 60%). The severity of depression in two groups is summarized in Table 1.

**Table 1:** Severity of diagnosed depression among study population

Severity of depression	Number of pseudoseizures patients	Number of control groups
Mild	8	8
Moderate	24	17
Severe	16	5
*No depression*	*2*	*20*

Depression was significantly more severe among patients with pseudoseizure (*P* < 0.0005). Severity of depression was not related to marital status or educational level in two groups (*P* = 0.342, 0.400). Most of women with pseudoseizure had moderate depression.

## DISCUSSION

Pseudoseizures are paroxysmal changes in behavior that bear a resemblance to epileptic seizures but are without any organic cause. They account for half of inpatient and one-fifth of outpatient epilepsy visits.[9–11] On the other hand, masked depression may increase the risk of somatization and may be hard for doctors to diagnose the problem.[12] It is common knowledge that many health professional dislike treating patients with somatoform disorders. The leading reason for this process is some doctors and the community (may be as a result of failing in educational system) do not believe/know that the condition of such a patient is beyond his /her control.[13]

Several risk factors for pseudoseizures have been described, including history of sexual/physical abuse, family or personal history of epilepsy, and prior head injury from which abuse was noted to be a high predilection for mood disorders as well.[14,15] On the other hand, the distinction between mental and physical illness points out that in a malady, both mind and body are involved.[16] To this may be added that the symptoms of depression include disorders of physical movements.[17] Patients with depression might have lower threshold for experiencing somatic symptoms in general.

Results of a large, cross-sectional medical outcome study showed that subjects with current depressive disorder or with only depressive symptoms displayed poorer physical and social performance when compared with healthy subjects.[18] Patients with somatoform disorders take their additional part in this process, because they believe in having a physical illness and do not seek psychiatric support.

In the present study, depression was common and more severe among patients with recently developed pseudoseizure compared to other neurologic disorders which urged patients to an emergency room. Findings suggest that, conversion disorder may be in part of a chronic and complex psychiatric process that extends transient somatoform symptoms.

The main limitation of our study is the diagnosis procedure. The evaluation of the patients by using standardized check lists during admission would made our results more acceptable; however, this tertiary hospital patients had the advantage of being visited by specialists and using all necessary laboratory tests to rule out differential diagnoses, which are usually clinical. Another limitation of this study is that the case selection is not population based. However, patients were included consecutively in this study from the main referral hospital in our region, which made them closer to a community sample than a sample from a psychiatric or specialist epilepsy center. We believe that the results will be useful while considering the characteristics of the study population.

Our findings are in accordance with studies reporting a high prevalence of psychiatric comorbidity in patients with pseudoseizure.

Our study clearly demonstrates that comorbid depression is typical for new cases of pseudoseizure, indicating that it is not persistent illness that produces depression in this group. Multiple psychiatric comorbidity is associated with increased functional impairment. Our findings underline the need for a rather comprehensive and broad treatment approach to pseudoseizure patients. Integrated evaluation and treatment approach including both neurologic and psychiatric resources are clearly needed.
